# Implementing psychosocial interventions within low and middle-income countries to improve community-based care for people with psychosis—A situation analysis

**DOI:** 10.3389/fpsyt.2022.807259

**Published:** 2022-08-01

**Authors:** Victoria Jane Bird, Syjo Davis, Abeer Jawed, Onaiza Qureshi, Padmavati Ramachandran, Areeba Shahab, Lakshmi Venkatraman

**Affiliations:** ^1^Unit for Social and Community Psychiatry, Wolfson Institute of Population Health, Queen Mary University of London, London, United Kingdom; ^2^Schizophrenia Research Foundation, Chennai, India; ^3^Interactive Research and Development, Karachi, Pakistan

**Keywords:** severe mental illness, psychosis, psychological interventions, India, Pakistan, low and middle-income countries, situation analysis

## Abstract

**Background:**

Globally, a treatment gap exists for individuals with severe mental illness, with 75% of people with psychosis failing to receive appropriate care. This is most pronounced in low and middle-income countries, where there are neither the financial nor human resources to provide high-quality community-based care. Low-cost, evidence-based interventions are urgently needed to address this treatment gap.

**Aim:**

To conduct a situation analysis to (i) describe the provision of psychosocial interventions within the context of existing care in two LMICs-India and Pakistan, and (ii) understand the barriers and facilitators of delivering a new psychosocial intervention.

**Method:**

A situation analysis including a quantitative survey and individual interviews with clinicians, patients and caregivers was conducted. Quantitative survey data was collected from staff members at 11 sites (private and government run hospitals) to assess organizational readiness to implement a new psychosocial intervention. To obtain in-depth information, 24 stakeholders including clinicians and service managers were interviewed about the typical care they provide and/or receive, and their experience of either accessing or delivering psychosocial interventions. This was triangulated by six interviews with carer and patient representatives.

**Results and discussion:**

The results highlight the positive views toward psychosocial interventions within routine care and the enthusiasm for multidisciplinary working. However, barriers to implementation such as clinician time, individual attitudes toward psychosocial interventions and organizational concerns including the lack of space within the facility were highlighted. Such barriers need to be taken into consideration when designing how best to implement and sustain new psychosocial interventions for the community treatment of psychosis within LMICs.

## Introduction

It is estimated that 5–8% of the world's population suffer from severe mental illness (SMI) (1). Despite its prevalence, the majority of individuals with SMI, do not have access to appropriate and effective community-based care (2, 3). Within low and middle-income countries (LMICs), an estimated 69–89% of people with SMI experience a treatment gap. This is most pronounced for psychosis, where 75% of all individuals fail to receive adequate care (4), despite a high financial burden and poor quality of life (5).

Approximately 20% of the world's population live in India and Pakistan, making it one of the most densely populated regions on the planet. The Global Burden of Disease study in 2017 estimated that 3.5 million people or 0.3% of the population in India have schizophrenia, with a further 7.6 million people (0.6% of the population) having bipolar disorder (6). Although estimating the percentage of people with psychosis in Pakistan is challenging due to the quality of data and a lack of electronic medical records in some regions, estimates suggest that approximately 1.5% of the population (3.3 million people) suffer from schizophrenia or related disorders (7).

Community-based psychosocial interventions are recommended in the treatment and management of psychosis across different high-income countries (8), as they have been shown to be effective in reducing symptoms, relapse and hospital admission rates, and improving functioning (9). A systematic review and meta-analysis that specifically focused on community-based psychosocial interventions within LMICs, mirrored these findings. However, only two of the included studies were conducted in India and none were included from Pakistan (7). Since the review, there has been further evidence of the positive impact of psychosocial interventions on outcomes for people with psychosis (10) including in India (11, 12) and Pakistan (13, 14) whilst small-scale studies have indicated that community-based family intervention can be feasible and beneficial for patients with depression and anxiety (15). Despite, these positive outcomes, treatment for people with psychosis within India and Pakistan predominantly consists of antipsychotic medication and tends to center on inpatient and hospital-based services (7).

Within LMICs such as India and Pakistan, there are limited financial and human resources to deliver psychosocial interventions, especially those specialist interventions that require certain professionals and/or competencies. Low-intensity interventions developed in high-income countries have not been routinely implemented or thoroughly tested within different LMIC contexts. Approaches that can be used by a range of staff within routine settings, without the need for expensive new services, have been identified as a feasible and sustainable way of reducing the treatment gap (16).

PIECEs is a National Institute of Health Research (NIHR-funded) Research and Innovation for Global Health Transformation (RIGHT) programme (NIHR200824) that aims to improve community based care for people with psychosis in India and Pakistan. The programme is working with key stakeholders including patients with psychosis, family and caregivers and clinicians to adapt an existing low-cost, generic and evidence-based intervention called DIALOG+ (17–20) so it can be feasibly applied within Pakistan and India to improve the quality of life for individuals with chronic psychosis.

DIALOG+ is a psychosocial intervention, which aims to make the routine meetings between clinicians and patients with mental illness, therapeutically effective. The intervention is delivered via an App on a tablet computer or smartphone and is based on the principles of quality of life, patient-centered consultations and solution focused therapy. DIALOG+ consists of a patient-centered assessment whereby patients rate their satisfaction with eight different life domains and three treatment aspects on a tablet computer. This is followed by a four-step solution-focused approach to identify their resources and develop solutions to deal with the concerns. The intervention has been shown to be effective in improving quality of life and reducing symptoms in a range of RCTs conducted in both high income countries (HICs) and LMICs, including the UK, Austria, Uganda, Colombia, and within five South-Eastern European countries (14, 21–23). With only minimal training (around 90 min), different staff members who have clinical contact with individuals with mental illness can be trained to deliver the intervention. Therefore, it is generic and requires neither specialist professionals, nor new services and referrals to bring about therapeutic change. Instead, it works by empowering patients to undertake actions to address their own concerns (24).

To adapt any psychosocial intervention, such as DIALOG+, it is vital to first understand existing services, assess stakeholder readiness, and determine any contextual barriers and facilitators of implementation. Consequently, the aim of this study was to conduct a situation analysis of the provision of existing psychosocial interventions for individuals with psychosis practiced in a range of settings within two urban areas of India and Pakistan. The investigation explored organizational and individual patient, caregiver and clinician-level barriers and facilitators of implementing a new psychosocial approach.

## Methodology

### Design

We conducted a situation analysis across two urban sites in India and Pakistan, utilizing both a quantitative survey (called the structured site visit form) and qualitative interviews with mental health providers, which were validated by people with psychosis and their caregivers.

### Setting

This situation analysis took place in Chennai, India and Karachi, Pakistan. Government and non-government-run hospitals were included in each site, with a purposive sampling method used to identify services to be interviewed between March and April 2021. The Department of Psychiatry and Behavioral Sciences at the Jinnah Postgraduate Medical Centre (a public tertiary mental health facility) and Karwan-e-Hayat, a welfare-run mental health facility were included in data collection in Pakistan. In Chennai, the situation analysis was expanded to gather data on existing mental health services across the city. This included government run tertiary care hospital, single practitioner private psychiatric clinics, group psychiatric practices, general hospital psychiatry units, private psychiatric hospital, and non-government organizations.

### Participants

Participants for the two sections of the situation analysis varied. For the quantitative survey, the lead clinician and/or service manager was approached to complete the information. For inclusion in the quantitative survey, participants were required to have (i) worked for a service providing care to people with psychosis and (ii) have an overview of the services provided within their organization. Lead clinicians and service managers were then asked to identify other clinicians in their service for the qualitative interviews with key informants. Additionally, clinicians from the different services were approached to take part in individual interviews. To validate the service provider interviews, the Lived Experience Advisory Panel (LEAPs) set up at for each site as part of the PIECES research project were used to identify eligible patient and caregiver representatives.

To be eligible, patients were required to (i) have current or previous lived experience of psychosis, (ii) have used mental health services within one of the included organizations, (iii) be aged 18 years and older, (iv) have capacity to provide informed consent and (v) be willing and able to discuss their experience of care with a researcher.

### Materials

For the quantitative survey, the pre-designed structured site readiness tool was used. The tool has been previously used in global health studies evaluating the use of psychosocial interventions in routine care across different LMICs (25–27). The questionnaire is designed to assess the readiness of an organization to implement a new psychosocial intervention. It includes items on the following: (1) organizational structure of local services, (2) type and number of patients seen, (3) type and number of employed clinicians, (4) care components typically delivered, and (5) practical structural information regarding the physical environment and financial mechanisms underpinning the services. A full copy of the form is shown in Supplementary material 1.

The site readiness tool was complemented by semi-structured interviews with clinicians and service managers to capture in-depth, rich descriptions of the care provided and types of psychosocial interventions delivered within routine services. The topic guides for the clinician interviews with clinicians explored the following topics: (i) service structure and function, (ii) typical care provided to patients, (iii) experience of delivering existing psychosocial interventions, and (iv) barriers and facilitators including organizational readiness to adopt new psychosocial approaches. The topic guide was developed through consensus discussions within the research team, which includes individuals from India, Pakistan and the UK. The topic guide was adapted for use with patients and carer as part of the validation interviews, and to ensure appropriate language was used within each site.

### Procedure

Included services were identified by the principal investigators at each site, based on previous experience with health facilities within the area and their professional networks. Following identification of a service, the lead clinician and/or service manager in charge of the services was approached to complete the structured questionnaire. All participants completed the pre-designed tool after signing the informed consent document. Each participant was also asked to respond qualitatively to the items in the structured tool.

Key clinicians within each healthcare facility, were identified through in-person snowballing techniques. Initially, the lead clinicians were asked to identify potentially eligible staff. Following the initial clinician interviews, staff, and members of the LEAPs in each site helped identify patients and caregivers to act as representatives for the validation interviews. Following completion of the informed consent form, researchers completed an in-depth interview. The interviews were conducted in English and Tamil by SJ, PR and LV in the Chennai site and in Urdu by AJ, AS and OQ in the Karachi sites. All interview facilitators received prior training for qualitative methods and data collection techniques. Researchers came from a range of backgrounds including health services research, psychiatry, psychology, and social work. The interviews were conducted in a quiet private space, were audio-taped, transcribed verbatim and translated into English as required.

In accordance with national COVID-19 guidelines, interviews were conducted through online conferencing software such as Zoom, Skype and WhatsApp and lasted between 40 and 60mins. Data saturation was defined when no new information was added to the codebook.

### Data analysis

Basic descriptive statistics for participant characteristics (including gender and staff designation) and facility details (such as number of presenting patients each year, number of facility staff, availability of clinical supervision and opportunities for CPD) were collected through the structured site visit form, supplemented by interviews, and collated through Microsoft Excel. Additionally, basic demographic information was collected as part of the semi-structured interviews. In all cases, descriptive statistics including means and ranges were used to summarize the data.

For the qualitative data analysis, individual interviews were transcribed, translated and anonymized by each site before sharing. As the interviews were primarily interested in the experience of delivering and/or receiving psychosocial interventions rather than in-depth accounts of mental distress, the topic guide was used as an initial guide to develop the themes. After data immersion, two interviews were coded to inform a codebook developed by PR and shared with five members of the research team for review and iterations (PR, OQ, SD, LV, VB). The initial codebook was used for coding an additional four interviews by two members of the research team (OQ and SJ). Following a process of coding comparison and discussion to address areas of divergence, a final version of the coding framework was developed and used to code the remaining interviews on QSR NVivo.

### Ethics

Ethical approval for this study was sought under the PIECEs programme. Ethical approval for the overall project was granted by Queen Mary University of London's Research Ethics Committee (QM28_10_20) and local ethics approval granted by IRD International Review Board in Karachi, Pakistan (IRD_IRB_2021_01_005) and the Schizophrenia Research Foundation's Ethics Committee in Chennai, India (SRF-CR/14/OCT-2020).

## Results

### Sample characteristics

A sample of 11 facilities (nine in Chennai, two in Karachi), which varied from large government run hospitals through to single private psychiatric practices, completed the quantitative structured questionnaire. Twenty-four individual interviews were conducted with a range of clinicians from 11 different mental health care facilities. The interviews explored existing practices for mental health treatment within healthcare systems across Chennai, India and Karachi, Pakistan. The data from the structured questionnaire has been integrated alongside the themes from the individual interviews, which explored the areas included in the questionnaire in more depth. The characteristics of individuals who took part in the interviews are shown in Table 1. Additionally, the themes from the clinician interviews were triangulated by interviews with three caregivers and three people with psychosis, who took part as patient representatives. Demographics of the patient and carer representatives are shown in Table 2.

**Table 1:** Socio-demographic profile of staff members interviewed at the selected facilities.

	**India**	**Pakistan**
Mean age (yrs)	47.7	49.5
Sex		
Male	5	5
Female	11	3
Mean duration of experience (yrs)	17.7	15.9
Types of participants	1. Psychiatrists 2. Psychologists 3. Social Workers 4. Management staff	1. Psychiatrist 2. Psychologist 3. Management staff
Type of organization	1. NGO 2. Pvt. Psychiatrist 3. Pvt. Psychiatric hospital 4. Pvt. GHPU 5. Pvt. Psychiatrist Group Practice 6. Govt. tertiary care hospital	1. NGO 2. Govt. tertiary care hospital

**Table 2:** Socio-demographic profile of patient and caregiver participants.

	**India**	**Pakistan**
Participant type		
Persons with mental illness	2	1
Carer	2 (father, brother)	1 (mother)
Sex		
Male	4	0
Female	0	2
Mean age of participants (yrs)	57	59.5
Mean duration of illness (yrs)	13.75	28.5

Data from the structured questionnaires and themes from the individual interviews were organized using a thematic approach into the following overarching framework: (i) existing practice (ii) barriers and facilitators of implementing a new psychosocial intervention and (iii) organizational readiness to adopt a new approach. A full coding framework is shown in Supplementary material 2.

### Existing practices

#### Contact with the mental health services

There was significant variance in the number of patients reported across facilities with large-scale hospitals or charity-run organizations seeing higher patient volumes compared to private practices (Table 3). The average duration of contacts varied depending on whether the contact was for a new or returning patient, with patient follow-up appointments shorter than the initial assessments. Duration of contact also varied by facility and was dependent on patient load, specific needs of the patient and caregivers and the setting e.g., whether in-patient admission, an OPD walk-in or planned appointment.

**Table 3:** Description of patient volumes, average duration of contact, frequency of contact, decision making by type of facilities.

	**NGOs** **(****3****)**	**Private psychiatric facility** **(****6****)**	**Government hospitals** **(****2****)**
Number of patients seen in a week	281	64	2250
Average duration of each contact	10 mins−1 h	10 mins−30 mins	10–30 mins
Frequency of contact	1 week−6 months	1 week−6 months	1 week−1 month
Individuals initiating treatment decisions	Clinicians	Clinicians and caregivers	Clinicians

Across both settings, a range of psychosocial interventions were implemented, although patients varied as to whether they had access. The different types of services offered are outlined in Table 4.

**Table 4:** Summary of types of services offered to people with severe mental illnesses by type of mental health facility in the study sample.

**NGOs** **(****3****)**	**Private psychiatric facility** **(****6****)**	**Government hospitals** **(****2****)**
Psychological Services e.g., CBT, family interventions, art therapy, psychoeducation, pharmacological therapy with psychiatric management, ECT, Vocational therapy, rehabilitation services, in-patient admission, long term care, community outreach, psychiatric management, out-patient services	Psychological services e.g., psychoeducation, pharmacological therapy with psychiatric management, ECT, vocational therapy, rehabilitation wards, in-patient admission, long term care, referrals to other specialists for comorbid conditions or halfway homes, tele counseling, job placements, government welfare schemes, specialist mental health treatments e.g. transcranial stimulation and ketamine transfusion	Psychological services e.g., CBT, social skills training, pharmacological therapy with psychiatric management, ECT, vocational therapy, rehabilitation services, in-patient admission

#### Multidisciplinary working

In both countries, the majority of patients were seen by multiple mental health professionals, with an emphasis on providing an integrated multidisciplinary team. Within government /NGO services, the care pathway appeared to follow a flow from the reception (first point of contact) through to the case manager/trainee/nurse and then to the consultant psychiatrist. In contrast, in private clinics, the psychiatrist or the psychologist directly handled the contact from the off.

“*We have Social workers, general doctors, clinical psychologists, rehab practitioners, occupational therapists, community nurses, community mobilisers, community psychologists and community psychiatrist, child psychologists, psychiatric nurses - all of these people work collaboratively to make up the core mental health team”. [Head of medical services, NGO, Karachi, Pakistan]*

Across the sites, facility staff worked together as a team in a collaborative, integrated fashion to address the needs of patients and caregivers holistically. For example, the nurse manages their physical assessment, case-manager and psychologist their psychosocial assessment, and psychiatrist focuses on medication management.

“*Ward psychologists, OPD psychologists, day care and rehab psychologists discuss patient treatment plans as a team and communicate with the psychiatrist whenever required. So in an integrated manner we are providing all the services to the patients” (Head of Medical Services in NGO, Karachi, Pakistan)*.

Despite a lower number of allied health professionals, clinicians reported that a wide range of services were offered to individuals with psychosis (as shown in Table 4). These included medical consultations, psychosocial interventions, and psychological therapies. In addition, support services in the form of free medications, in-patient care when required, day care programmes and employment services were available in some services. This was corroborated in the individual interviews with patients also reporting a wide variety of services including non-pharmacological interventions, psychological therapies and structured psychosocial interventions. However, pharmacological interventions were still the most common form of treatment available to most patients, especially those in government run facilities, who were able to provide subsidized medicines to patients.

#### New cases

In both locations, the procedure for registering new cases involved a range of staff including case managers, social workers and consultants who work together to take the patient's physical and mental history and evaluate their treatment needs. Special care is given to the needs identified by the patient, or their caregivers and psychoeducation is provided at this stage with the aim of increasing awareness and treatment compliance.

The duration of sessions for new cases depends on symptom severity and can take anywhere between 10 and 90 min (with the average estimated at 30 min). Many clinicians report that decisions around treatment options are generally clinician-led. Clinicians are aware of this responsibility, noting that caregivers and patients depend heavily on them for directing priorities and managing care. Some clinicians also see this as an opportunity to build rapport with family members to support the patient's on-going management at home.

“*Patients and families they sort of surrender all responsibilities of what needs to be done” (Psychiatrist, NGO, Chennai, India)*.

“*We spend a lot of time with relatives, especially primary caretakers. So we cashed into the Indian social, system where the parents and or, or the family plays a very, very vital role supporting the patient. And that family support is pretty strong. So we try to cash in on it as much as possible. So a lot of emphasis from our side is to work with the family” (Psychiatrist, Private practice, Chennai, India)*.

#### Follow-up

Patients are often called back to the facility for review on a fortnightly basis. Frequency of contact between patients and clinicians varied from 1 week to 6 months in India and 1 week to 1 month in Pakistan. In many facilities, the clinicians who initiated assessment and treatment for the patient remain consistent for their review, to enable the relationship to form and to maintain continuity of care – essential in the delivery of many psychosocial interventions. Another approach taken by some facilities, seen most commonly in Pakistan where medication is heavily subsided or free, is to link the quantity of medication prescribed so that it finishes by the next follow-up. This helps to encourage patients to come back to check in with their clinicians. Clinicians tend to increase the time (from 1 week to 6 months) between review sessions, and some offer other contact methods if the patient is managing their condition well-or lives far away.

“*I see the patient today, probably after a week, then afterwards, I will see them after about a week and a half, then probably a month, then may be it is quite stable month and a half, 40 days” (Psychiatrist, Private practice, Chennai, India)*.

The proportion of patients presenting with psychosis or psychosis-related conditions varied in the facilities from 30 to 45%. However, respondents in larger facilities reported that the majority of their cases (80%) present with severe or chronic mental health conditions and are typically admitted for inpatient care. Some of these patients belong to a lower socio-economic status and cannot afford mental health care. Clinicians within these facilities noted that included among those admitted were people who may be homeless, those with a long duration of untreated psychosis and individuals who have already experienced multiple types of medical and non-medical interventions.

“*I can give you an example of a lady who kept a tub in her room and passed stool in that, because she was very scared of going to the washroom, because she had hallucinations. Her husband thought that it was the doing of some supernatural being [possessed] or somebody had done magic on her. So up until 2 years ago she was just being treated for magic and from faith healers. So the patients […], estimated they come to us after 10 years. […]. So by the time they come here, they have had such cognitive deterioration, and the symptoms are so great that that person takes more time, longer time to recover” (Clinical Psychologist and Rehab In-charge in NGO, Karachi, Pakistan)*.

#### Involvement in care and treatment decisions

Some clinicians identified a disagreement between their own treatment goals and those presented by patients and caregivers. Whilst there was a general perception of the benefits of caregiver and patient involvement in treatment planning, clinicians find it necessary and sometimes challenging to counsel their clients to re-prioritize their treatment goals. They reported that most of the concerns identified by patients and their caregivers related to physical, social or lifestyle challenges i.e., sleep issues, weight gain, sexual dysfunction or reproductive health, rather than mental health symptomatology.

“*Come in with acute relapse […] and that is the time when they suddenly come and ask we are planning for pregnancy can we go ahead with it? […] In the middle of the acute psychotic episode, those are the time we will have to put our foot down and say like your priorities are definitely not right” [Senior Psychiatrist, NGO, Chennai, India]*.

One clinician also identified a struggle to encourage the caregivers to support patients to try non-pharmaceutical interventions, including psychosocial interventions. A general focus on the medical model was identified as a barrier in changing attitudes toward integrating psychosocial approaches in treatment.

In the majority of cases, it is the clinician or other healthcare worker who initiates the discussion and advises treatment options. While there are accounts of clinicians involving both caregivers and patients in these sessions, generally, more importance was given to caregiver accounts of the patient's condition as clinicians identified issues around capacity and a reduction in cognitive functioning of patients. Only a handful of clinicians assigned significant value toward facilitating more independence by patients in directing their treatment plans, as it was perceived as developing motivation for self-care and management.

“*So then we sit down and then make a list of okay now you're presenting these things, how do you think we can help you and what are the things you want to work on first. […] Typically, ensuring that the client presents, just so that the motivation levels are more than us trying to tell them, I think it's better to start working on this”. - [Psychologist, Private practice, NGO, Chennai, India]*

This was triangulated by two of the caregivers interviewed, who felt they were actively involved to either verify the information relayed by the patient or to keep a check on them. However, involving caregivers in the long-run may not always be possible as often family members were only consulted once, right at the start of the treatment.

“*Initially in 2013 when we came to meet her they called me separately and asked few things and then my son separately and asked few things then they would make us sit together and ask few other things, but after that nothing of that sort has happened till date*.” [Caregiver in LEAP, Chennai, India]

#### Home visits

There were mixed responses to the system of home visits. In Chennai, while the NGO had a system in place for home visits, the private clinicians did not do any home calls and home visits were often limited to picking up patients for admission when family members made requests.

“*No we don't do home visits. Personally I don't do home visits, but patients and families do request for home visits. But nowadays what we have started is, we have… we will send the ambulance to bring a patient who is not willing for treatment and is violent, we'll send the staff nurse. If the male nurse is going to fetch the patient and if the patient is willing to talk to the male nurse, immediately a video call will be done and I will try to talk to the patient..” (Private Psychiatrist in Private Practice, Chennai, India)*.

### Barriers and facilitators of implementing a new psychosocial intervention

The second overarching theme related specifically to the barriers and facilitators that different stakeholders anticipated when delivering a new psychosocial intervention. These barriers and facilitators were reported in the individual interviews with clinicians and further verified by the patient and caregiver representatives. Barriers and facilitators existed and operated at different levels, from the individual patient level, through to organizational difficulties.

#### Accessing appropriate care

Although not reported directly by clinicians, during the triangulation interviews, caregiver and patient representatives frequently discussed issues accessing appropriate care. This included logistical difficulties such as the distance and cost of services, to the beliefs of patients and relatives.

“*Difficulties like costs of transportation, rent sometimes is a lot…she would insist on going in the rickshaw. So in the start I would take her in the rickshaw but then when coming back I would bring her in the bus…as its costing a lot of money like in rickshaws they used to ask us for a lot like 300..so yeah difficulties have come a lot in going to and fro (from the facility*)” [Caregiver in LEAP, Karachi, Pakistan].

“*My family they thought it was black magic, so they didn't allow me to take my sister out to hospital…my family had history of mental illness; they were also not getting treatment…so that was the first barrier for me my own family members won't allow me to take her out*” [Caregiver in LEAP, Chennai, India].

One patient revealed being ‘locked up' for 18 years due to a lack of awareness of mental health problems on the family's part. Another patient talked about their attendant making them discontinue medication.

Barriers that were specific to the patient's symptoms were a common theme. A caregiver in Karachi, discussed how their relatives were resistant to treatment follow-ups as it involved them waking up early to attend appointment which were mostly in the mornings. Another caregiver shared how their relative's cognitive issues, which can be compounded by facility wait times, meant patients could become difficult to manage.

“*I have to get her ready in half an hour by the time I reach [Facility] she becomes more dull…it takes at least 30 min to 1 h to see the doctor…it takes 30 min, that is in a free time, if there are lot of people coming in then it will take more than 30 min and she tends to get very fidgety to sit somewhere other than home she doesn't find comfortable*.” [Caregiver in LEAP, Chennai, India]

#### Use of technology

The increased use of technology, especially in service delivery at both sites, could overcome and address barriers faced when accessing care. In particular, technology facilitated maintaining contact with patients, following up on clinical status, including early identification of relapse, increased medication compliance, and has helped to deliver some interventions.

“*We use technology to maintain contact with the patient. To make sure that they take their medication in a timely manner, through their caregivers. How is the patient feeling, whether or not they are facing any relapse etc.” (Service Director and Management, CEO of NGO, Karachi, Pakistan)*.

Follow-up methods often increasingly made use of technology, for example, a respondent from India reported facilitating follow up via “*email communication”* (Lead Psychiatrist in Private Psychiatry Group Practice, Chennai, India).

Finally, within this theme, electronic medical record systems were identified as helpful in keeping track of review cases, with the exception of a government facility in Pakistan that depended entirely on paper-based records.

#### Funding availability

The availability of funding was reported to be a challenge in both sites. Whether an NGO in Chennai or the government run facility in Pakistan, limited availability of financial resources was seen to limit the scope of services provided, including the provision of psychosocial interventions. In a lot of cases, a nominal charge is paid by the family to access care, with families reporting that the cost of care was further compounded by the costs associated with travel.

“*As we operate on zakat and donation, the welfare department ascertains whether a patient can pay a certain part of their fees and then charges the patient according to affordability.” (Head of Medical Services of NGO, Karachi, Pakistan)*

#### COVID-19 pandemic

Clinicians across Pakistan and India reported a drastic drop in outpatient volume as well as admissions due to the COVID-19 pandemic. They attested to multiple challenges for patients seeking psychiatric help, ranging from a lack of public transport to reach facilities to the unavailability of psychiatric medication due to pharmaceutical companies shutting down or prioritizing the supply of other medicines. Clinicians also reported being cognisant of the fact that the pandemic had affected income for a large section of the population making it even more difficult to afford mental health services.

Numerous changes were introduced to the routine services including clinical contact with patients and caregivers and training opportunities for healthcare workers. Social distancing and the COVID measures put in place within services made it difficult to deliver many psychosocial interventions, which rely on extended face-to-face contacts.

“*We received instructions from Admin because a huge number of patients are there in the psychiatric OPD in our 2 OPD days. We were told that you have to see only 50 patients in a major OPD day. So it was difficult to see which 50 patients should be seen, should it be initial patients who came first or do we see patient's with needs like if they are aggressive or violent or unmanageable at home.” [Psychiatrist in Government hospital, Karachi, Pakistan]*

Alongside strict COVID-19 procedures including the use of masks, sanitiser, social distancing and screens between doctors and patients, clinicians reported shifting to prescribing cheaper and more readily available medicines, providing online and telephonic consultations, and ensuring that their patients are able to reach them via WhatsApp or email. However, these measures further hampered the delivery of psychosocial interventions, which were emphasized less compared to pharmacological approaches.

### Organizational readiness to adopt a new intervention

The final part of the framework relates to organizational readiness to adopt new psychosocial interventions and approaches within services. Data for this section of the framework was initially collected via free-text items on the structured site visit form and supplemented with interviews with clinicians to elaborate on the emergent themes. Table 5 provides a summary of the themes reported across both countries.

**Table 5:** Key findings from healthcare workers on barriers and facilitators to the adoption of a new intervention at mental health facilities in India and Pakistan.

**Themes**	**Key findings across sites**
Acceptance of adopting new interventions	- Lack of suitability to local context (PK^**^), personal willingness (IN^***^+PK) were among the factors contributing toward reluctance among individual clinicians to adopting new interventions + Belief in enhanced quality of consultation structure (IN), and flexibility to adapt and utilize new approaches (PK+IN) were among the factors facilitating willingness among individual clinicians to adopting new interventions
Time and resource constraints	- Facilities experience a constant under-staffing of human resources (IN) causing high caseloads and pressure on existing workforce (IN+PK) makes the buy-in of a new intervention challenging - With limited consultation time and a crowded facility setting, clinicians identified the lack of time as a major barrier to the utilization of a new intervention (IN+PK)
Structural factors	- Long waiting times and lack of appropriate space in facility for ensuring privacy or storage (IN+PK) - Limited funding from public-sector (IN) for human resources and equipment (computers and software) needed for adopting new interventions (IN+PK) + New interventions should be adapted to align within routine care, scheduled appointments (IN+PK) and among specific providers at facilities (IN)
Managerial support	+ Facility management's support is paramount for willingness of clinicians to adopt new interventions (IN+PK) - Need to change multiple areas and practices to accommodate a new intervention (IN+PK)
Use of technology	- Younger clinicians more likely perceived to adopt technology-mediated interventions and resistance expected from more senior consultants (IN) + Facility staff open to use of new technology-based approaches for mental health delivery (IN+PK) + Existing practices as COVID-19 prompted the use of technology in mental health delivery e.g. virtual consultation and online M&E systems for data security and privacy (IN)
Training in using new interventions	- Facilities do not have a separate training department or structured training process for new recruits to learn how to utilize interventions (IN) + Existing systems of support and supervision (e.g. by peer clinicians and senior consultants) at mental health facilities facilitates the adoption of new interventions (IN+PK)

** PK Findings from Pakistani participants.

*** IN Findings from Indian participants.

#### Buy in or acceptance of the interventions

Clinician attitudes could either be a barrier or a facilitator to successful implementation. A senior psychiatrist from Chennai felt that older psychiatrists might be reluctant to adopt a new intervention due to their existing experiences.

“*The first I think, its more a human barrier first I think if you ask me. In the sense people must be convinced that this is worth trying …….they could be sceptical about it right at the beginning you know. So it's a lot of attitude and as you said human barrier rather than managerial barrier or any other barrier so if you are able to get at least 50 to 60 % of the mental health professionals feel convinced about this and say ‘it okay let's see if it works or not',” (Psychiatrist in NGO, Chennai, India)*

This was reiterated by others, who stated that many clinicians are resistant to change and there is a need to be committed and flexible to implement a new program. In addition, there was a strong need to create awareness specifically about non-pharmacological approaches within services. This also extended to the patients and their families, and was corroborated by family members as being an important influence on their attitudes toward psychosocial interventions.

“*Awareness of non-pharmacological intervention is very very less like under their protection; my child or my daughter should come out of the symptoms, only the medicines will be sure is what they… Yeah front line of treatment is the medicines but alongside non-pharmacological intervention is also available is what we need to reintegrate, emphasize and repeatedly talk to them then only it will get registered” (Manager, NGO)*.

#### Case load and time constraints

High caseloads in all study sites were noted as a main barrier to the implementation of psychosocial interventions. In all public mental health facilities, it appeared that caseloads were high.

“*In an outpatient clinic to bring in a specialized intervention it is always going to be difficult because the number of patients who require services stays very high” (Psychiatrist in NGO, Chennai, India)*.

Linked to high caseloads, the time required to implement psychosocial interventions and the lack of time during routine consultations was reported by the majority of clinicians as a major barrier to changing practice. Particular issues were encountered where psychiatrists provided private consultations, it was felt that psychosocial interventions, could potentially eat into the paid consultations in their practice.

“*I think it is also a question of the amount of time that is being spent. So, again what will happen is if we are going to do this for a sub group of patients on any regular day, it may not be always possible to do it as part of the regular OPD, we will have to do on appointment on a separate day where there is no pressure of time or pressure of other patient….” (Management Staff in NGO, Chennai, India)*.

The time spent delivering interventions was felt to affect the running of a busy service. Instead, many felt that psychosocial interventions should be offered as a specialized intervention for a select population only. In acute presentations or in busy OPDs it was felt that a structured psychosocial intervention would be too difficult to deliver due to time and resource constraints, particularly in terms of sustained implementation.

“B*ut I always say this, I keep saying that at [Facility] there is extreme enthusiasm initially for everything, then after 3–4 months we wouldn't know what happens.” (Psychiatrist in NGO, Chennai, India)*.

Scheduling appointments ahead of time was seen as one way of overcoming issues of clinician availability.

“*Well, our schedule is very very tight and is difficult for us, but if there is training then we schedule our requirements and appointments beforehand to manage the time slot if we find out 2–4 days in advance. We schedule ourselves accordingly to manage.” (Clinical Psychologist in NGO, Karachi, Pakistan)*.

Within the triangulation interviews, caregivers overwhelmingly responded positively to accessing a new treatment method especially if it was recommended by their clinician or discussed with the care provider. Furthermore, one caregiver accepted a new intervention or treatment method even if it required more time since it can be an additional activity for the patients who otherwise are mostly homebound with limited activities.

“*I usually defer it to [doctor's name] if she says you should take it, i usually take it. I won't say no to her.” (Caregiver in LEAP, Chennai, India)*.

#### Structural factors at the facilities

Linked to the above, hospitals and OPDs appeared to be crowded, with a high number of patients accessing the facilities on an average day. Within this context, privacy was noted to be a challenge, and one that occurred across all facilities.

“*we are seeing a huge number of patients and it is very crowded. We have to examine patients in front of everyone, and there are usually two to three patients in the same room. We don't really have a choice in the matter as our existing set-up cannot accommodate the large volumes of patients […]Our current building is not sound-proof and does not account for any form of privacy for the patient.” (Psychiatrist, Government Hospital, Karachi, Pakistan)*.

Although patients and clinicians in some facilities stated that there was always space for private consultations, in many services more than one clinician shared the space, which raised issues concerning patient confidentiality. Often space within OPDs were not large enough for the delivery of separate psychosocial interventions.

“*we also simultaneously see two patients in one room – but those who need individual attention, psychosexual or private conversation, then they see a psychologist or transformation and within that set up we adjust them – we don't see every patient individually” (Psychiatrist and Dean, Dept of Psychiatry in NGO, Karachi Pakistan)*.

Even where private rooms were available for consultations, there were still issues surrounding privacy.

‘*'Yes, so we have separate rooms. They're not soundproof, but of course their voice cannot go outside the premises. We have two cubicles, but we have a library, and my office, basically in my office I take family intervention sessions. So that doesn't have an issue of confidentiality, but of course, in your case we have space.” (Clinical Psychologist and Rehab In-charge, NGO, Karachi, Pakistan)*.

#### Managerial support

Many respondents reported that management was supportive of implementing new approaches. However, a need for increasing clinician numbers, and managing the caseloads effectively was felt to be critical for successful implementation.

“*I don't think anything needs to be changed in the organization, each individual will have to plan out his/her work and it must work on with appointments, it may not work as part of regular OP service” (Psychiatrist in NGO, Chennai, India)*.

Many respondents felt that structural and functional changes were needed to accommodate new psychosocial interventions, like the DIALOG+ intervention within PIECEs. Clinicians suggested several areas where changes needed to be made to allow the seamless introduction and implementation of the intervention. One key suggestion was to only include patients who already have a good rapport with the clinician, stating these patients could initially be engaged in a novel intervention.

“*I feel like with a few, when they do get to know me better, I think it depends a lot on the client & the psychiatrist engagement- So once the engagement & the- what do you call that- rapport is there then using this might be helpful with clients.” (Psychiatrist in Private practice, Chennai, India)*.

#### Use technology and technology-related issues

The psychosocial intervention within the PIECEs project, DIALOG+, is delivered on a tablet computer. There were mixed responses to the use of a tablet for delivering a psychosocial intervention. While one senior psychiatrist felt it was a matter of getting used to using the device, several others pointed out challenges in the use of devices for delivering interventions. These included the need for training and supervision, software related issues, difficulty in documenting on a device, internet connectivity and cost of devices.

“*I think it is more to do with the internet bandwidth available, and some of the software that we are using can be little difficult to use and so making… no keeping records on that particular format in the software is a little bit stressful, actually it's not little bit stressful its quite stressful” (Psychiatrist in NGO, Chennai, India)*

Another respondent implied the difficulty in using a device when the caseload was high. Furthermore, many clinicians reported still preferring paper and pen format over a device, especially older clinicians.

#### Training in using new psychosocial interventions

The majority of interviews with clinicians and service managers in the NGO and Governmental organizations across both countries identified that facilities typically had structures in place for the continued professional development of their workers. These structures could be utilized in the event of capacity-building required for the delivery of a new intervention. This included residency programs for teaching hospitals, skills training for nurses, sessions for psychologists and social workers on counseling and communication, workshops on specific psychiatric topics or research methodologies. Training was either delivered in house or externally at conferences. Due to COVID-19, some of the facilities reported a reduction in the frequency of these trainings but there was general consensus on the benefit of structured capacity-building for a variety of medical staff at the facilities as well as being trained in the use of a particular psychosocial approach (Clinical Psychologist, NGO, Karachi, Pakistan) (Management Staff in NGO, Chennai, India).

“*So that is amazing, the whole of the clinical team will be available for this training. Because to some extent every clinician is interacting with patients so there is a definite need for constant training. Why not from a versatile trainer too. We can make it possible, you arrange it and we will definitely ensure that all our clinicians attend” (Head of Medical Services, NGO, Karachi, Pakistan)*.

Supervisory mechanisms for the evaluation and quality control of mental health service provision was available at all facilities interviewed with the exception of two NGOs and one private practice group in India.

“*I am not very sure if the consultants themselves are getting supervised I don't think we have any regular audits or anything about how a particular patient or how a particular consultant is managing, because I think it is more an individual consultant or his or her own way of managing clients.” (Psychiatrist in NGO, Chennai, India)*.

In addition to organizational supervision being provided e.g., as part of a residency program or clinical practice, a handful of clinicians identified that peer supervision to troubleshoot issues related to complicated treatment protocols amongst the mental health workers in a facility was an enabler in supporting organizational readiness.

“*Well like, like you have your peers in your department, you might not have them in one department but we still we still have a tightly knit circle of, you know, psychiatrist you know we who commonly, you know, share our challenges and the problems that we face” – [Psychiatrist, Private practice, Chennai, India)*.

## Discussion

The situational analysis aimed to understand the provision of psychosocial interventions within the context of existing services in the two study sites in India and Pakistan, and explore the readiness to integrate a new technology-based psychosocial intervention.

A diverse range of mental health care facilities were included in the study. There was an overall consensus on the benefits of taking a collaborative and integrated approach when delivering mental health care to patients with psychosis. This approach has been recognized as vital to the long-term successful and holistic management of the treatment needs of people with severe mental health conditions in a variety of contexts (28). Within the services included in the study, psychiatrists typically referred individuals in need of psychosocial therapies to psychologists, occupational and rehabilitation therapists, where available, as these staff members could spend more time with patients and their caregivers to resolve social concerns and provide psychoeducation for on-going self-care and management.

Although staff, patients and caregivers were positive about psychosocial interventions, there were a number of practical and organizational barriers to their routine delivery. In particular, the time taken to deliver a psychosocial intervention and the impact this would have on the rest of the caseload was a primary concern. This was especially the case for large government run hospitals and outpatient departments (OPDs) where staff were expected to see large volumes of patients per day, and typically could only spend 10 min per patient. In such services, pharmacological approaches tended to dominate.

The use of technology both to support and deliver new psychosocial interventions was seen as a barrier and facilitator, with some clinicians raising concerns about technical issues, whilst others stated that technology, including electronic medical records, could support an integrated approach to mental health care.

### Strengths and limitations

The study had a number of strengths. Firstly, it recruited a range of different services, which varied in their size and funding mechanisms from single psychiatrists in private practices to large government run hospitals. Secondly, a mixed methods approach was used, with findings from the quantitative survey corroborated and expanded upon by in-depth individual interviews. The interviews with clinicians were further triangulated with data from patient and caregiver representatives. Thirdly, a wide range of clinician participants were included such as service managers, psychiatrists, social workers and nurses. This enabled us to understand the current provision of psychosocial interventions from multiple perspectives.

Despite these strengths, there were three main limitations. The selected services included in the study were from two cities, Chennai and Karachi, and were a self-selected sample. Services across India and Pakistan may vary in terms of their approach to the care of people with psychosis. However, in this case, the decision was taken to focus on services in the regions where the intervention within the PIECEs programme will be initially implemented in order to understand the local context for service delivery and help us develop tailored implementation plans. Finally, although used as a method for triangulation and validation, only six patient and caregiver participants were included in the project. The included individuals were patient representatives identified via the project's existing lived experience advisory panels. A decision to use only patient and caregiver representatives was taken, in part, due to the difficulties in accessing groups of individuals during the COVID-19 pandemic. Despite the low numbers, the interviews produced rich descriptions that corroborated and expanded upon many of the themes identified by the clinicians. Future studies could include a larger sample of patient and caregiver participants.

### Clinical and research implications

In both India and Pakistan there is a dearth of specialized mental health professionals, with a range of 0.25–0.75 psychiatrists for every 100,000 individuals in the population (29, 30). Psychiatrists within the study viewed their workload as a barrier to addressing the psychosocial concerns raised by their patients and caregivers. If a holistic approach that integrates both psychosocial and pharmacological management is to be integrated into this workforce, system strengthening, capacity-building, and managerial changes to build human resources is required. This includes the introduction of collaborative approaches with primary health care facilities to reduce the institutional burden attributed to mental health problems within low resource settings (31, 32).

A few clinicians in the included facilities acknowledged the importance of utilizing a patient-centered model and actively engaged their patients to contribute toward their treatment priorities and goals. However, it was often felt that patient concerns differed from the clinical symptom management goals set by the clinicians. Furthermore, the majority of clinicians demonstrated resistance in relying on the account of patients with psychosis as they did not consider them to have enough insight and capacity to be independently involved. In recent years, normative guidelines, including those published by the Lancet Global Health Commission on High-Quality Health Systems (33), and the World Health Organization underscore the importance of patient-centered approaches in health systems in recognition that ‘people have the right to receive dignified and respectful care that is responsive to their needs' (34). While this approach is still in formative stages in LMICs, there is strong evidence to suggest that mental health services that are patient-centered and responsive to individual needs are linked to improved contact rates with mental health services, better treatment compliance and a high level of patient satisfaction. Given that many people with psychosis in LMICs struggle with continuity of care, social disempowerment and access barriers (35), integrating a holistic and patient-centered psychosocial response to psychiatric management within facilities in India and Pakistan has the potential to strengthen mental health response and make mental health more equitable and responsive to patient needs.

Clinicians highlighted a need for increased community awareness of mental health, arguing that awareness raising does not stop at providing patients with proper mental health care and support, but should cover the importance of continuity of care. A lack of awareness at the community and family level can lead to worsening treatment prospects and outlook. Since the majority of the clinicians report involving caregivers, psychosocial interventions that include a component that actively engages support systems from caregivers and other people around the patient's family can be well-received. A previous study has shown that family psychoeducation that aims to improve the caregiver's insight into the illness, enabled both the family and the patient to cope in a more effective way and resulted in a significant improvement in overall quality of life scores (36). More recent studies related to employment of persons with psychosis also point to the need of involving families in the provision of vocational rehabilitation services (37, 38). As seen in the interviews by caregivers, buy-in for existing or new interventions is heavily dependent on the clinicians who manage their relative's condition. Based on this evidence, it is worth ensuring that facility-based psychosocial interventions, in South Asian contexts, should involve a multi-collaborative stakeholder approach and target clinicians, caregivers and patients together to improve acceptability and access to support. In addition, interventions should also factor in the caregiver's needs due to the additional burden of care on them and consider integrating access that limits the cost of transport to facilities.

Contrary to popular belief, patients with psychosis in both Pakistan and India attested to a variety of mental health services being accessible for them, although this varied across institutions. Additionally, most patients, if not all, confirmed that they were easily able to get in touch with their clinicians via email or text for advice on medicines or management of symptoms – a service that has undoubtedly helped many during the COVID-19 pandemic. However, it must be noted that individuals included within the study, were already in contact with services. Further work may be required to include the perspectives of individuals with mental distress who are not in contact with mental health services.

Although there was a clear want and need for psychosocial interventions, expressed by clinicians and then validated by patients and caregivers, a range of structural organization and personnel related barriers to their sustained implementation were highlighted in both the quantitative survey and the interviews. The range of barriers suggests that merely focusing on the cultural competence and sensitivity of a new psychosocial intervention to be implemented will not be sufficient. Instead, projects aiming to implement new approaches need to consider the local context, and especially any context specific barriers, when designing both the intervention and implementation plans.

## Conclusion

Across the different services and settings for the interviews, there was consistent support for the use of psychosocial interventions to treat patients with psychosis in a multidisciplinary and integrated approach. However, there is a need to address organizational level barriers, mainly surrounding the lack of human resources across mental health services in India and Pakistan. Any new psychosocial will need to develop implementation and awareness strategies in a multi-level approach, targeting patients, caregivers, clinicians and the organization as a whole. Additionally, there is a need for local mental health services that incorporate the expectations of caregivers of people with mental health conditions to foster better coordination between stakeholders, and enhance support for reintegration into the community, and ultimately recovery for individuals.

## Data availability statement

The raw data supporting the conclusions of this article will be made available by the authors, without undue reservation.

## Ethics statement

The studies involving human participants were reviewed and approved by Queen Mary University of London and the Ethics Committees of Schizophrenia Research Foundation and Interactive Research and Development. The patients/participants provided their written informed consent to participate in this study.

## Author contributions

VB and PR were responsible for obtaining the original funding for the project. All authors contributed to the design, conduct, analysis, and write up of the manuscript.

## Funding

This study was funded by the National Institute for Health Research (NIHR) via its RIGHT Programme [Improving outcomes for people with psychosis in Pakistan and India- enhancing the Effectiveness of Community-based care (PIECEs)] Grant number NIHR200824, using UK aid from the UK Government to support global health research.

## Conflict of interest

Authors AJ, OQ, and AS were employed by Interactive Research and Development. The remaining authors declare that the research was conducted in the absence of any commercial or financial relationships that could be construed as a potential conflict of interest.

## Publisher's note

All claims expressed in this article are solely those of the authors and do not necessarily represent those of their affiliated organizations, or those of the publisher, the editors and the reviewers. Any product that may be evaluated in this article, or claim that may be made by its manufacturer, is not guaranteed or endorsed by the publisher.

## Author's disclaimer

The views expressed are those of the author(s) and not necessarily those of the NIHR or the Department of Health and Social Care.
