# Supplementary material for: Implementing psychosocial interventions within low and middle-income countries to improve community-based care for people with psychosis—A situation analysis
Source: Front Psychiatry. 2022 Aug 1;13:807259. doi: 10.3389/fpsyt.2022.807259 (PMC9376469; doi:10.3389/fpsyt.2022.807259)
Supplement: Supplementary file 1 [file Data_Sheet_1.DOCX]

**Supplement 1. STRUCTURED VISIT FORM**

**CLINICAL SITE**

**DIALOG+**

| **Site/Institution name:** | | **Address:** |
| --- | --- | --- |
| **Date of Visit** (dd/mm/yy) | **Name of the person from the coordinating site conducting the visit:** | |

| **Principal Investigator (PI)’s Information** | | | |
| --- | --- | --- | --- |
| Name of the PI |  | | |
| Title |  | | |
| Email |  | | |
| Telephone |  | Fax: | |
| **Clinical Staff present at the site visit** | | | |
| **Name of the Clinical Staff** | | | **Role *(psychiatrist/psychologist/nurse)*** |
|  | | |  |
|  | | |  |
|  | | |  |
|  | | |  |
|  | | |  |
|  | | |  |
|  | | |  |
|  | | |  |

1. **SERVICE DESCRIPTION**

| 1. **How many patients with severe mental illness in total are seen every year in the service?** | | |
| --- | --- | --- |
| *(Please specify a number)* | | |
| Comments: | | |
| 1. **How many clinical staff are working in the service?** | | |
| (Please specify a number) | | |
| Comments: | | |
| - 1. **Among the clinical staff working in the service, how many are:** | | |
| Psychiatrists | |  |
| Psychologists | |  |
| Other psychotherapists | |  |
| Nurses | |  |
| Social workers | |  |
| Occupational therapists | |  |
| Other mental health professionals (please specify) | |  |
| Comments: | | |
| 1. **Are patients in the service usually seen by the same clinician for routine meetings?** | | |
| *If yes – what type of clinician* | | |
| *If no – how many clinicians does the patient typically see for routine meetings* | | |
| Comments: | | |
| 1. **On average, how often are patients with severe mental illness seen by the clinician for a routine meeting? E.g. once per month, once per week** | | |
| Please specify a number: | | |
| Comments: | | |
| 1. **How long does a routine meeting with a patient generally last?** | | |
| Please specify minutes: | | |
| Comments: | | |
| 1. **What type of treatment does the service offer to patients with severe mental illness?** | | |
| ◻ | Medication | |
| ◻ | Psychotherapy (i.e. Cognitive Behavioural Therapy, **Family Intervention**, other psychotherapy approaches) | |
| ◻ | Occupational therapy | |
| ◻ | Home visits | |
| ◻ | Other (please specify: Social worker and employment advice______________) | |
| **Please write any further comment about Service Description in the space below** | | |
|  | | |

1. **FACILITY ASSESSMENT**

| 1. **Has the site dedicated storage (i.e. locked cabinet) to securely store study materials (i.e. tablets)** | | **YES** | **NO** |
| --- | --- | --- | --- |
| *If No, please specify:* | | | |
| Comments: | | | |
| 1. **Is there internet (WiFi) facility available at the site?** | | **YES** | **NO** |
| *If No, please specify:* | | | |
| Comments: | | | |
| 1. **Is a printer available at the site?** | | **YES** | **NO** |
| Comments: | | | |
| 1. **Is there equipment available for photo copying and scanning?** | | **YES** | **NO** |
| Comments: | | | |
| 1. **Where will routine meetings with patients participating in the study be conducted?** | | | |
| ◻  ◻  ◻  ◻  ◻ | Patient’s home  Community Mental Health Centre (CMHC)  Hospital outpatient department  Both patient’s home and CMHC  Other (please specify: _________________________________________________________________) | | |
| Comments: | | | |
| 1. **Has the site dedicated space for conducting routine meetings with patients participating in the study?** | | | |
| If No, please specify: | | | |
| Comments: | | | |
| 1. **Has the site dedicated space to conduct training for clinicians?** | | | |
| If No, please specify: | | | |
| Comments: | | | |
| 1. **How does the site store patient medical records?** | | | |
| ◻ Electronically  ◻ Hard copies  ◻ Both | | | |
| Comments: | | | |
| **Please write any further comment about Facility Assessment in the space below** | | | |
|  | | | |

1. **ORGANIZATIONAL READINESS**

| 1. Are there any barriers (i.e. managerial, structural) to the implementation of the project in the clinical service as proposed? | **YES** | **NO** |
| --- | --- | --- |
| Comments: | | |
| 1. Do clinicians have regular supervision with their line manager? | **YES** | **NO** |
| If yes, please specify how often: | | |
| Comments: | | |
| 1. Do clinicians undertake continuous professional development training? | **YES** | **NO** |
| If yes, please report some examples of such activities: | | |
| Comments: | | |
| **Please write any further comment about Organization Readiness in the space below** | | |
|  | | |

1. **CLINICAL STAFF INVOLVED IN THE STUDY**

| **Clinical Staff** | **Professional background** | **Years of experience in mental health** | **Intention to stay in the current position for 3 months** | **Number of patients with severe mental illness regularly seen monthly** | **Line manager** | **Present during site visit** |
| --- | --- | --- | --- | --- | --- | --- |
|  |  |  | YES ◻  NO ◻ |  |  | YES ◻  NO ◻ |
|  |  |  | YES ◻  NO ◻ |  |  | YES ◻  NO ◻ |
|  |  |  | YES ◻  NO ◻ |  |  | YES ◻  NO ◻ |
|  |  |  | YES ◻  NO ◻ |  |  | YES ◻  NO ◻ |
|  |  |  | YES ◻  NO ◻ |  |  | YES ◻  NO ◻ |
|  |  |  | YES ◻  NO ◻ |  |  | YES ◻  NO ◻ |
|  |  |  | YES ◻  NO ◻ |  |  | YES ◻  NO ◻ |
|  |  |  | YES ◻  NO ◻ |  |  | YES ◻  NO ◻ |
|  |  |  | YES ◻  NO ◻ |  |  | YES ◻  NO ◻ |
|  |  |  | YES ◻  NO ◻ |  |  | YES ◻  NO ◻ |
|  |  |  | YES ◻  NO ◻ |  |  | YES ◻  NO ◻ |
|  |  |  | YES ◻  NO ◻ |  |  | YES ◻  NO ◻ |
|  |  |  | YES ◻  NO ◻ |  |  | YES ◻  NO ◻ |
|  |  |  | YES ◻  NO ◻ |  |  | YES ◻  NO ◻ |
|  |  |  | YES ◻  NO ◻ |  |  | YES ◻  NO ◻ |
|  |  |  | YES ◻  NO ◻ |  |  | YES ◻  NO ◻ |
|  |  |  | YES ◻  NO ◻ |  |  | YES ◻  NO ◻ |
|  |  |  | YES ◻  NO ◻ |  |  | YES ◻  NO ◻ |

____________________________________________________________

(Signature of the person conducting the visit)

______________________________________________________________

(Signature of the Principal Investigator at the site)
