# Supplementary material for: Implementing psychosocial interventions within low and middle-income countries to improve community-based care for people with psychosis—A situation analysis
Source: Front Psychiatry. 2022 Aug 1;13:807259. doi: 10.3389/fpsyt.2022.807259 (PMC9376469; doi:10.3389/fpsyt.2022.807259)
Supplement: Supplementary file 2 [file Data_Sheet_2.DOCX]

Supplement 2: Coding framework

| **Theme** | **Sub-theme** | **Sub-theme** |
| --- | --- | --- |
| Existing practices | Contact with mental health services | Access to existing psychosocial interventions |
|  | Multidisciplinary working | Integrative and collaborative care |
|  |  | Range of services on offer |
|  |  | Dominance of medication services |
|  | New cases | History taking |
|  |  | Whole-team approach |
|  |  | Provision of psychoeducation |
|  | Follow up | Frequency of contact |
|  |  | Determined by severity of presenting problem |
|  |  | Continuity of care |
|  | Involvement in treatment and care decisions | Disagreement between patient and clinician |
|  |  | Role of caregivers in supporting patients |
|  |  | Initiation and discussions during the appointment |
|  |  | Decisions regarding initiation of psychosocial interventions |
|  | Home visits |  |
| Barriers and facilitators | Accessing appropriate care | Distance to care facilities |
|  |  | Cost of accessing services (out of pocket expenses) |
|  |  | Beliefs of patients and relatives |
|  |  | Condition specific barriers such as cognition or motivation |
|  | Use of technology | Technology to overcome distance and travel requirements |
|  |  | Use of technology to enable frequent follow-ups |
|  |  | Access to technology |
|  |  | Increased use of technology during the pandemic |
|  | Funding | Limited financial resources as a barrier to access |
|  |  | Cost of travel was an additional financial burden |
|  | COVID-19 | Reduction in outpatient numbers |
|  |  | Lack of public transport |
|  |  | Social distancing requirements |
|  |  | Medication availability |
| Organisational readiness | Buy in or acceptance | Changes in existing processes |
|  |  | Procedures to introduce new treatments |
|  |  | Suitability to the local context |
|  |  | Enhanced quality of care |
|  |  | Incentives to change practice |
|  | Case load and time constraints | Waiting times |
|  |  | Understaffing |
|  |  | Pressure on existing staff |
|  | Structural factors | Physical environment |
|  |  | Space and privacy issues |
|  |  | Alignment with existing practices |
|  |  | Availability of funding |
|  | Managerial support | Managerial commitment to supervision |
|  |  | Fit with current procedures and structures |
|  | Technological related issues | Experience level of the clinicians |
|  |  | Covid-19 increasing need for new technologies |
|  |  | Willingness to adopt new technology |
|  | Training | Professional development |
|  |  | Supervision |
|  |  | Lack of training department or resources |
